# Influence of long term administration of tofogliflozin on chronic inflammation of visceral adipose tissue in mice with obesity induced by a high-fat diet

**DOI:** 10.1371/journal.pone.0211387

**Published:** 2019-01-25

**Authors:** Kohsuke Shirakawa, Wataru Yano, Keisuke Inoue, Yoshinori Katsumata, Jin Endo, Motoaki Sano

**Affiliations:** 1 Department of Cardiology, Keio University School of Medicine, Tokyo, Japan; 2 Tokyo New Drug Research Laboratories, Kowa Company, Ltd., Tokyo, Japan; Universidade do Estado do Rio de Janeiro, BRAZIL

## Abstract

We previously found that senescence of cluster of differentiation 4 (CD4) T cells is accelerated in the visceral adipose tissue (VAT) of mice with diet-induced obesity (DIO) due to a high-fat diet (HFD), and that these senescent-associated T cells cause chronic inflammation of visceral adipose tissue through secretion of osteopontin, provoking systemic insulin resistance. In this study, we examined whether the development of chronic inflammation and senescence-associated T cells in VAT of DIO mice was improved by long-term weight loss after switching to normal chow (NC) or by administration of a sodium glucose cotransporter 2 inhibitor (tofogliflozin). Wild-type mice were fed an HFD for 26 weeks from 4 weeks old. At 30 weeks of age, half of these DIO mice were switched to NC with or without 0.005% tofogliflozin for 38 weeks. The other mice remained on the HFD with or without 0.005% tofogliflozin for 38 weeks. When DIO mice were switched to NC, their weight decreased to that of mice kept on NC since weaning. After 38 weeks (68 weeks of age), chronic inflammation of the VAT subsided with disappearance of senescence-associated T cells. In the HFD groups, the carbohydrate intake per mouse was half or less of that in the NC group, and urinary glucose excretion by the effect of tofogliflozin was lower in the HFD mice than in the NC mice. Mice that remained on the HFD showed no improvement in chronic inflammation in VAT, possibly because urinary glucose excretion was not sufficiently promoted by tofogliflozin due to the low carbohydrate intake. Thus, no improvement in glucose metabolism or weight loss was observed in these mice.

## Introduction

Accumulation of visceral fat causes hypertension, diabetes mellitus, and dyslipidemia, leading to the development of cardiovascular disease, chronic kidney disease, or cancer over time [1–6]. These processes associated with the metabolic syndrome are also called the metabolic domino effect [7]. In addition to chronological aging, the acceleration of aging associated with the metabolic syndrome is called metabo-aging [7]. We previously found that senescence of immune cells is involved in the mechanism by which accumulation of visceral fat causes metabolic syndrome and/or metabo-aging [8].

Among the various immune cells, T cells are the most susceptible to the effects of aging [9]. With aging, cluster of differentiation 4 (CD4) T cells show functional abnormalities, or the acquired immune response to microorganisms decreases and excessive inflammatory reactions develop. These changes are caused by the increase in dysfunctional CD4 T cells among the total CD4 T cell population rather than by an overall decrease in CD4 T cells or decreased overall T cell function. These T cells cannot act effectively to regulate the immune system due to a reduced ability to produce cytokines. Instead, these T cells constantly secrete an inflammatory substance called osteopontin [10] [11]. Under normal circumstances, osteopontin is only produced when necessary and is involved in various physiological processes, such as modulation of tissue architecture and wound healing [12]. Constant production of osteopontin by these T cells causes chronic inflammation and/or pathological tissue remodeling [8] [10] [11]. An epidemiological study showed that the blood level of osteopontin was correlated with the prevalence of aging-related diseases such as cardiovascular disease and cardiac failure [13]. These osteopontin-producing T cells that are characterized by increased expression of programmed death-1 (PD-1). Although PD-1 is considered to be an immunosuppressive receptor, PD-1 stimulation does not inhibit osteopontin secretion [8][10]. T cells with this senescence-associated secretory phenotype are thought to trigger autoimmune responses or systemic inflammation that is a characteristic of the elderly. Accordingly, these CD4 T cells are also called senescence-associated T (SA-T) cells [8].

We found SA-T cells in the visceral adipose tissue (VAT) of mice with diet-induced obesity (DIO) due to a high-fat diet (HFD), and demonstrated that these SA-T cells provoke chronic inflammation in intra-abdominal fat by secretion of osteopontin, thus causing systemic insulin resistance [8]. SA-T cells showed high expression of γH2AX, a marker of DNA damage, and senescence-associated beta-galactosidase (SA-βgal), a marker of cellular aging. These findings suggested that SA-T cells are possibly involved in aging, not only associated with advancing chronological age but also with visceral fat obesity.

Can SA-T cells that develop in the visceral fat in association with obesity be removed by weight loss? To answer this question, we established DIO mice by feeding them an HFD until 30 weeks of age post-weaning and then switched these animals to normal chow (NC). After switching from the HFD to NC, food intake showed a transient decrease and the mice lost weight. While their food intake soon returned to normal, the lower body weight was maintained and the visceral fat and liver weight decreased to the same level as in mice fed only NC post-weaning. However, after 2 months of weight reduction, crown-like structures (a histopathological manifestation of chronic inflammation) were still present in VAT, the number of SA-T cells per gram of tissue was increased, and the blood osteopontin level remained high. These results suggested that SA-T cells persist in VAT for some time after weight reduction and that the effect of obesity persists (negative legacy effect) [14]. It has been reported that even if insulin resistance improves after weight loss, the risk of cardiovascular events is not reduced in humans [15]. A possible reason for the risk of cardiovascular events not falling after weight reduction may be that SA-T cells persist in VAT for a long time and continue to produce osteopontin [14].

Sodium glucose cotransporter 2 (SGLT2) inhibitors are a new class of oral antidiabetic agents that reduce the blood glucose level by inhibiting glucose reabsorption in the proximal renal tubules. These agents are commonly used by young obese patients with type 2 diabetes because weight reduction can be expected as calories are lost when glucose is excreted in the urine.

Against this background, the present study was performed to examine whether the development of chronic inflammation and SA-T cells of VAT in DIO mice (fed an HFD until 30 weeks of age post-weaning) was improved by long-term weight loss after switching to NC or by treatment with an SGLT2 inhibitor.

## Materials and methods

### Reagent

Tofogliflozin was provided by Kowa Company, Ltd. (Tokyo, Japan). 0.005% (w/w) of tofogliflozin were mixed with HFD (D12492, 60 kcal%fat, Research Diets, New Brunswick, NJ, USA) or NC (D12450J, 10 kcal%fat), and fed to mice about 4 mg/kg/day or 3 mg/kg/day, respectively. These were pharmacological doses of tofogliflozin [16][17] in mice.

### Animal care

Male C57BL/6J mice were purchased from Charles River Laboratories Japan (Yokohama, Japan). The mice were housed in an animal room under a 12-h light-dark cycle and allowed free access to food. Mice were fed with an HFD (D12492, 60 kcal% fat, Research Diets, New Brunswick, NJ, USA) from 4 weeks of age to create DIO mice. At 30 weeks old, the DIO mice were divided into four groups by using the SAS System (Release 9.4, SAS Institute Japan, Tokyo, Japan) combined with Stat Preclinica Client (Release 2.2, Takumi Information Technology, Tokyo, Japan) user interface statistical software on the basis of the body weight and other parameters. These groups (n = 10 each) were (1) the control diet group (NC group), (2) the control diet with 0.005% tofogliflozin group (NC + tofo group), (3) the HFD group, and (4) the HFD with 0.005% tofogliflozin group (HFD + tofo group). Briefly, half of the established DIO mice were switched to NC (D12450J 10 kcal% fat) at 30 weeks of age with or without 0.005% tofogliflozin (NC and NC + tofo groups) for 38 weeks. The other half of the HFD mice remained on the HFD with or without 0.005% tofogliflozin (HFD and HFD + tofo groups) for 38 weeks. The body weight and food intake were measured twice a week. Animal care and the experimental procedures were approved by the Animal Care Committee of Tokyo New Drug Research Laboratories at Kowa Company (approval no.: W609147).

### Biochemical parameters

Hemoglobin A1c (HbA1c) was measured by the DCA 2000 system using the DCA 2000 HbA1c cartridge (Siemens Healthcare Diagnostics, Tokyo, Japan) at 43 weeks of age. At 44 weeks of age, blood was collected from all animals following an overnight (16 h) fast and feeding for 4 h. Non-fasting blood samples were also collected in the morning at 52 weeks of age. For measurement of plasma glucose, triglycerides (TG), total cholesterol (TC), aspartate transaminase (AST), alanine transaminase (ALT), creatinine (CRE), and non-esterified fatty acid (NEFA) levels, the Qualigent GLU, Qualigent TG, Qualigent CHO, Qualigent AST, Qualigent ALT, Qualigent CRE, and Clinimate NEFA (Sekisui Medical, Tokyo, Japan) reagents were respectively used with a Labospect 003 Hitachi Automated Analyzer (Hitachi High-Technologies Corporation, Tokyo, Japan). Plasma levels of insulin (Shibayagi, Gunma, Japan) and adiponectin (Otsuka Pharmaceutical, Tokyo, Japan) were measured with ELISA kits, and 3-hydroxybutyrate was measured using an assay kit (3-HB-L, Kainos Laboratories, Tokyo, Japan). For 18-hour urine collection, individual mice were placed in metabolic cages with access to water and food at 30, 39, and 54 weeks of age. Urine glucose and creatinine levels were measured with Qualigent GLU and Qualigent CRE by the Hitachi Automated Analyzer. Urinary albumin was measured by the DCA 2000 system using a DCA 2000 Microalbumin/Creatinine reagent cartridge (Siemens Healthcare Diagnostics, Tokyo, Japan).

### Glucose and insulin tolerance tests

We performed an oral glucose tolerance test (OGTT; administration of 1.5 g/kg of glucose after 16 hours of fasting) and an insulin tolerance test (ITT; administration of 0.75 U/kg of insulin i.p. after 4 hours of fasting) to assess glucose intolerance and insulin resistance, respectively.

### Histological and immunological analyses

All mice were euthanized at 68 to 70 weeks of age for histological and immunological analyses. As previously reported [18], we stained and examined whole mounts of adipose tissue. Mice were sacrificed by cervical dislocation, after which the VAT was removed by a sterile technique and minced into small pieces (~2–3 mm) with a scalpel. The tissue pieces were washed, fixed in cellFIX (Cat. 340181, BD) for 60 min, and permeabilized with 0.1% Triton X-100 for 10 min. Then the specimens were blocked with 5% bovine serum albumin, incubated with the primary antibody [F4/80 (BM-8, eBioscience, San Diego, CA, USA)] overnight at 4°C, and then incubated with the Alexa Fluor 488-conjugated secondary antibody (Molecular Probes, Eugene, OR, USA) for 1 h. The tissues were counterstained for 1 h with BODIPY 558/568 (Molecular Probes) to visualize adipocytes and with 4',6-diamidino-2-phenylindole (DAPI; Molecular Probes) to visualize nuclei. For confocal microscopy (LSM 710, Carl Zeiss, Jena, Germany), the tissue samples were illuminated with four laser lines (405 nm, 488 nm, 568 nm, and 800 nm) and emissions were collected through appropriate narrow band-pass filters, after which images were acquired and processed by LSM 710 software.

### Isolation of the stromal vascular fraction and flow cytometry

We isolated stromal vascular cells by the method described previously [18], with some modifications. After systemic heparinization, mice were sacrificed under general anesthesia and the VAT was removed and ground into small pieces. Then samples were incubated for 40 min in collagenase II/DNase I solution (1 mg/ml collagenase II and 50 μg/ml DNase I in Hanks’ balanced salt solution) with gentle stirring, after which the digested tissue samples were centrifuged at 1000×g for 10 min. The resulting pellet was washed twice with cold phosphate-buffered saline and filtered through a 70-mm mesh. Red blood cells were lysed in erythrocyte lysis buffer (Biolegend, San Diego, CA, USA) for 10 min and the cells were resuspended in RPMI-1640 medium supplemented with 10% fetal bovine serum. Single-cell suspensions of the adipose stromal vascular fraction were blocked with CD16/32 monoclonal antibody (93; Biolegend) at 4°C for 5 min, followed by staining with a mixture of antibodies at 4°C for 20 min. Flow cytometric analysis and cell sorting were carried out with a FACSAriaIII (BD Biosciences) and data were analyzed by using FlowJo software (Tree Star). The following antibodies were employed: anti-CD3 (14A-2; Biolegend), anti-CD4 (GK1.5; Biolegend), anti-CD8a (OKT8; Biolegend), anti-CD11b (M1/70; Biolegend), anti-F4/80 (BM8; Biolegend), anti-PD-1 (29F.1A12, Biolegend), anti-CD19 (6D5, Biolegend), and anti-CD153 (RM153, Biolegend).

### Real-time quantitative PCR

Total RNA samples from sorted cells, and adipose tissue were prepared using an RNeasy Mini Kit (Qiagen) or Trizol reagent (Invitrogen), according to the manufacturer’s instructions. A First-Strand cDNA Synthesis kit (Invitrogen) was used for cDNA synthesis. Quantitative real-time PCR was performed using ViiA 7 Real-Time PCR System. GAPDH gene was used as an endogenous control to normalize for differences in the amount of total RNA in each sample. All values were expressed as fold increase or decrease relative to the expression of GAPDH. Primer sequences for genes are as follows: *Gapdh*, 5′-AGGTCGGTGTGAACGGATTTG and 3′-TGTAGACCATGTAGTTGAGGTCA; *Spp1*, 5’-CCCGGTGAAGTGCTGATT and 3′-TTCTTCAGAGGACACAGCATTC; Tnf, 5′-CCCTCACACTCAGATCATCTTCT and 3′-GCTACGACGTGGGCTACAG.

### ELISA

Levels of OPN (R&D systems), IFN-γ (Biolegend), IL-17 (R&D systems), and total IgG (eBiosciences) in supernatants or serum were determined by ELISA according to the manufacturers’ instructions.

### Statistical analysis

Results are presented as the mean ± standard error of the mean. The significance of differences among multiple groups was determined by performing non parametric test followed by or Bonferroni test. In all analyses, *P* <0.05 was considered significant.

## Results

**Fig 1:**
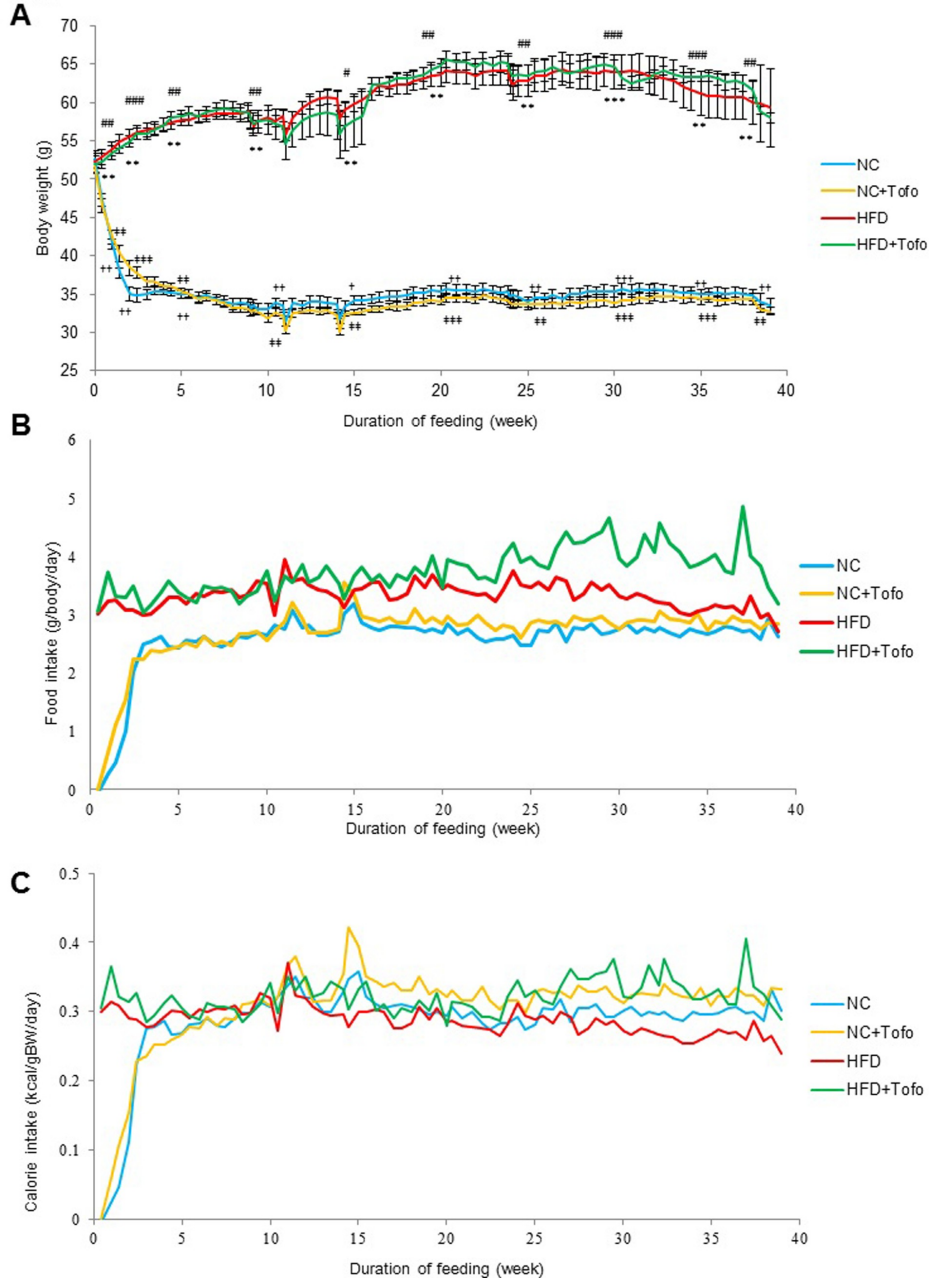
Weight and food intake of mice fed normal chow or a high-fat diet with/without tofogliflozin. Mice were fed an HFD for 26 weeks from 4 weeks of age. At 30 weeks of age, half of the mice were switched to normal chow (NC) with or without 0.005% tofogliflozin (NC and NC + tofo, respectively) for 38 weeks. The remaining HFD mice received the HFD with or without 0.005% tofogliflozin (HFD and HFD + tofo, respectively) for 38 weeks. (A) Body weight (n = 7–10 mice per group), (B) Food intake calculated from 2 cages per group, (C) calculated calorie intake from 2 cages per group Data represent the mean. Statistical analysis of body weight at 1, 2, 5, 10, 15, 20, 25, 30, 35, and 38 weeks of administration by the non-parametric Bonferroni test. **P<0.01, and ***P<0.001 (NC vs. HFD), #P<0.05, ##P<0.01 and ###P<0.001 (NC+Tofo vs. HFD+Tofo), †P<0.05, ††P<0.01, and †††P<0.001 (NC vs. HFD+Tofo), ‡‡P<0.01, ‡‡‡P<0.001 (NC+Tofo vs. HFD), There were neither significant differences between NC and NC+Tofo groups, nor between HFD and HFD+Tofo groups.

When DIO mice fed an HFD until 30 weeks of age were switched to NC, their food intake calculated from two cages per group tended to decrease transiently and their weight also declined (Fig 1A and 1B). Although the calorie intakes rapidly returned to the same as those of HFD (Fig 1B and 1C), the body weight after weight loss was maintained. Adding 0.005% tofogliflozin to NC did not affect the food intake or body weight. When mice remained on the HFD, their body weight continued to increase until it reached a plateau around 50 weeks of age. Adding 0.005% tofogliflozin to the HFD did not affect the food intake or body weight of these mice until 50 weeks of age (Fig 1A and 1B).

**Fig 2:**
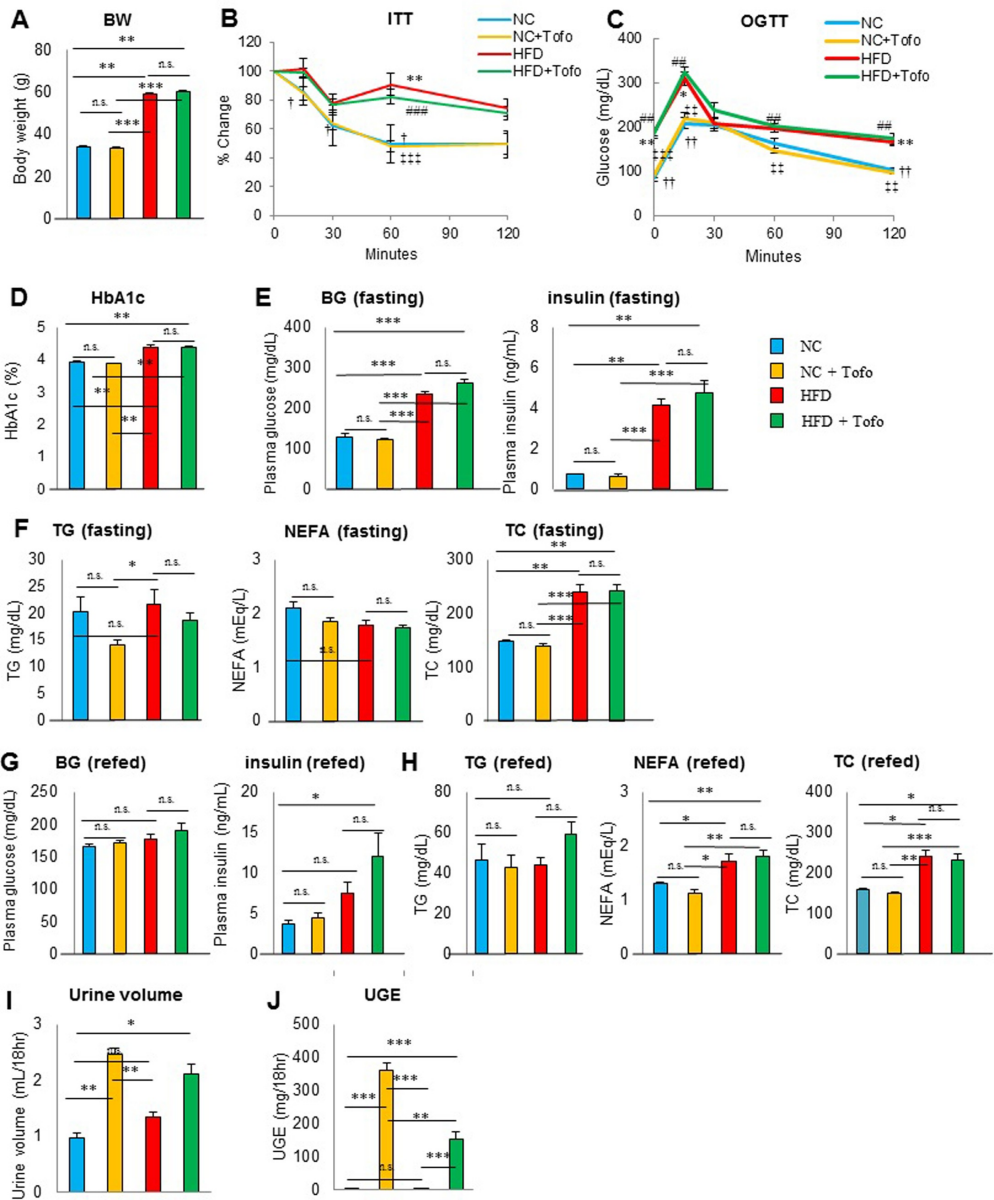
Effects of tofogliflozin in mice fed normal chow or a high-fat diet at 39–44 weeks of age. (A) Body weight at weeks of age, (B) Insulin tolerance test (ITT) at 40 weeks of age, and (C) oral glucose tolerance test (OGTT) at 41 weeks of age. (D) HbA1c at 43 weeks of age. (E) Glucose, insulin and (F) triglycerides (TG), non-esterified fatty acids (NEFA), and total cholesterol (TC) in 16 hr-fasting blood samples at 44 weeks of age. (G) Glucose, insulin, and (H) TG, NEFA, and TC in non-fasting blood samples at 44 weeks of age. (I) Urine volume, and (J) urinary glucose excretion (UGE) at 39 weeks of age. Data are the mean ± SEM (n = 9–10). *P < 0.05, **P < 0.01, and ***P < 0.001; NS: not significant by the non-parametric Bonferroni test.

An insulin tolerance test (40 weeks of age, at 10^th^ week of administration) and an oral glucose tolerance test (OGTT; 41 weeks of age, at 11^th^ week of administration) were performed in 10-11^th^ weeks of 0.005% tofogliflozin administration. The urine output and urinary glucose excretion were also measured. Decrease in body weight (Fig 2A), improvement in insulin sensitivity (Fig 2B) and improvement in glucose tolerance (Fig 2C) were noted in mice switched to the NC compared with mice remaining on the HFD. HbA1c, fasting blood glucose, insulin levels and TC were significantly improved compared to the HFD (Fig 2D–2F), however there were no significant difference in non-fasting glucose and insulin level (Fig 2G). On the other hand, non-fasting TC significantly improved after switching to the NC (Fig 2H). In mice switched to the NC, addition of tofogliflozin significantly increased urine output (Fig 2I) and urine glucose excertion (UGE) (Fig 2J), however these did not affect the body weight, food intake, insulin sensitivity, or glucose tolerance compared to the NC group. In the HFD, addition of tofogliflozin increased UGE, but did not reduce the body weight or improve insulin sensitivity or glucose tolerance. Furthermore, UGE of the HFD group withadding tofogliflozin was lower than that of the NC group adding tofogliflozin (Fig 2J).

**Fig 3:**
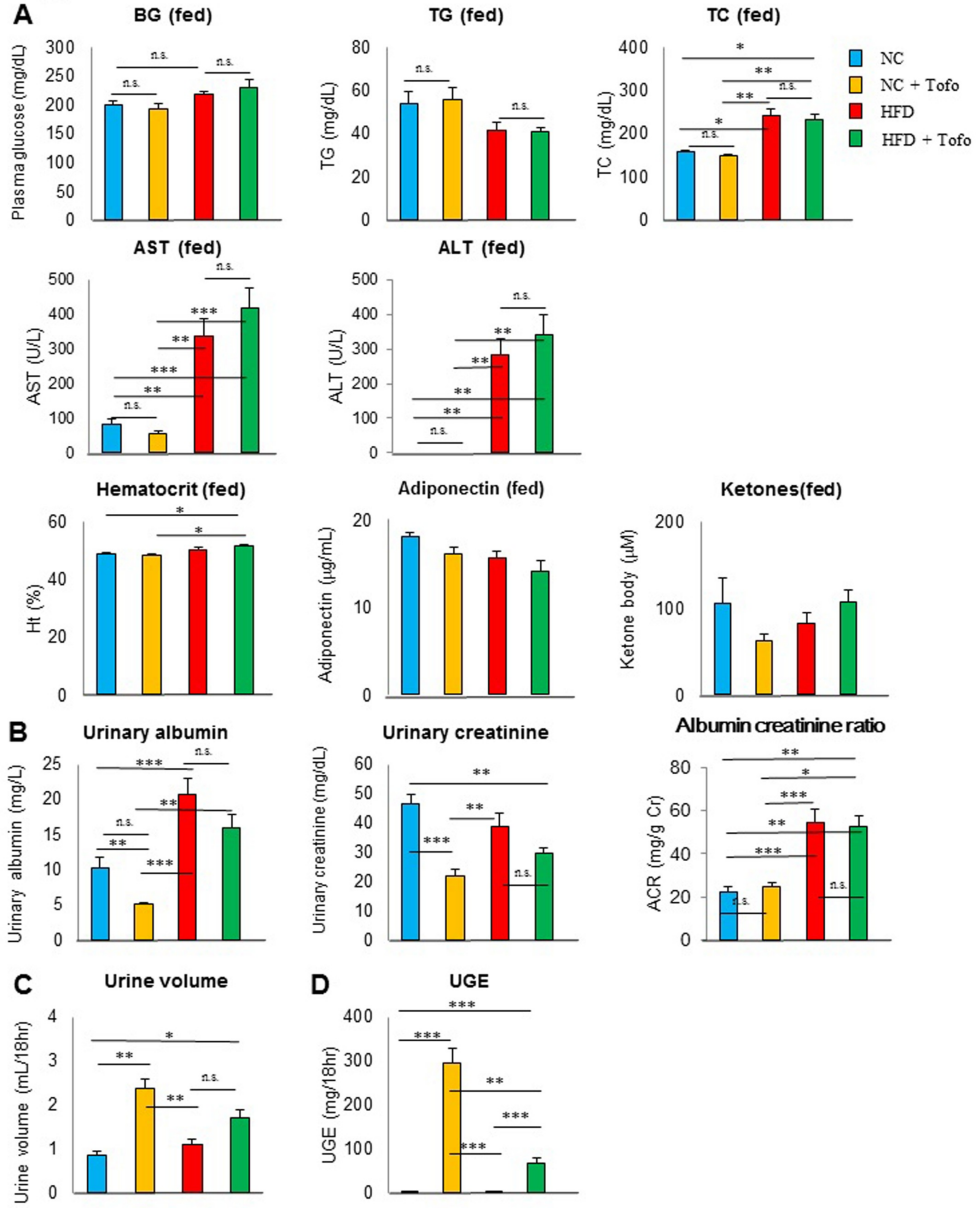
Effects of tofogliflozin in mice fed normal chow or a high-fat diet at 52–54 weeks of age. (A) Non-fasting glucose, TG, TC, aspartate transaminase (AST), alanine transaminase (ALT), hematocrit, adiponectin, and 3-hydroxybuthylate (ketone bodies) in the fed state at 52 weeks of age. (B) Urinary albumin, urinary creatinine, and albumin/creatinine ratio (ACR), (C) urine volume, (D) UGE, at 54 weeks of age. Data are the mean ± SEM (n = 8–10). *P < 0.05, **P < 0.01, and ***P < 0.001; NS: not significant by the non-parametric Bonferroni test.

The experiment was continued to investigate the longer-term effects of tofogliflozin. In 22^th^ week of administration (52 weeks of age), the non-fasting blood glucose of mice on the HFD was the same as that of mice switched to NC. TC, aspartate transaminase (AST) and alanine transaminase (ALT) were higher, and adiponectin tended to be lower in the HFD group compared to those in the switching to the NC group (Fig 3A). Adding tofogliflozin to the diet did not affect the non-fasting blood glucose, TG, TC, AST, ALT, adiponectin, ketone bodies, or hematocrit (Ht). At 24^th^ week of administration (54 weeks of age), the urine albumin-creatinine ratio of mice on the HFD was higher than that of mice switched to NC (Fig 3B). There was no significant difference in urine output and UGE between these groups (Fig 3C and 3D). In the HFD groups, addition of tofogliflozin to the HFD increased UGE (Fig 3D), however which did not result in the improvement in the albumin-creatinine ratio (Fig 3B).

**Fig 4:**
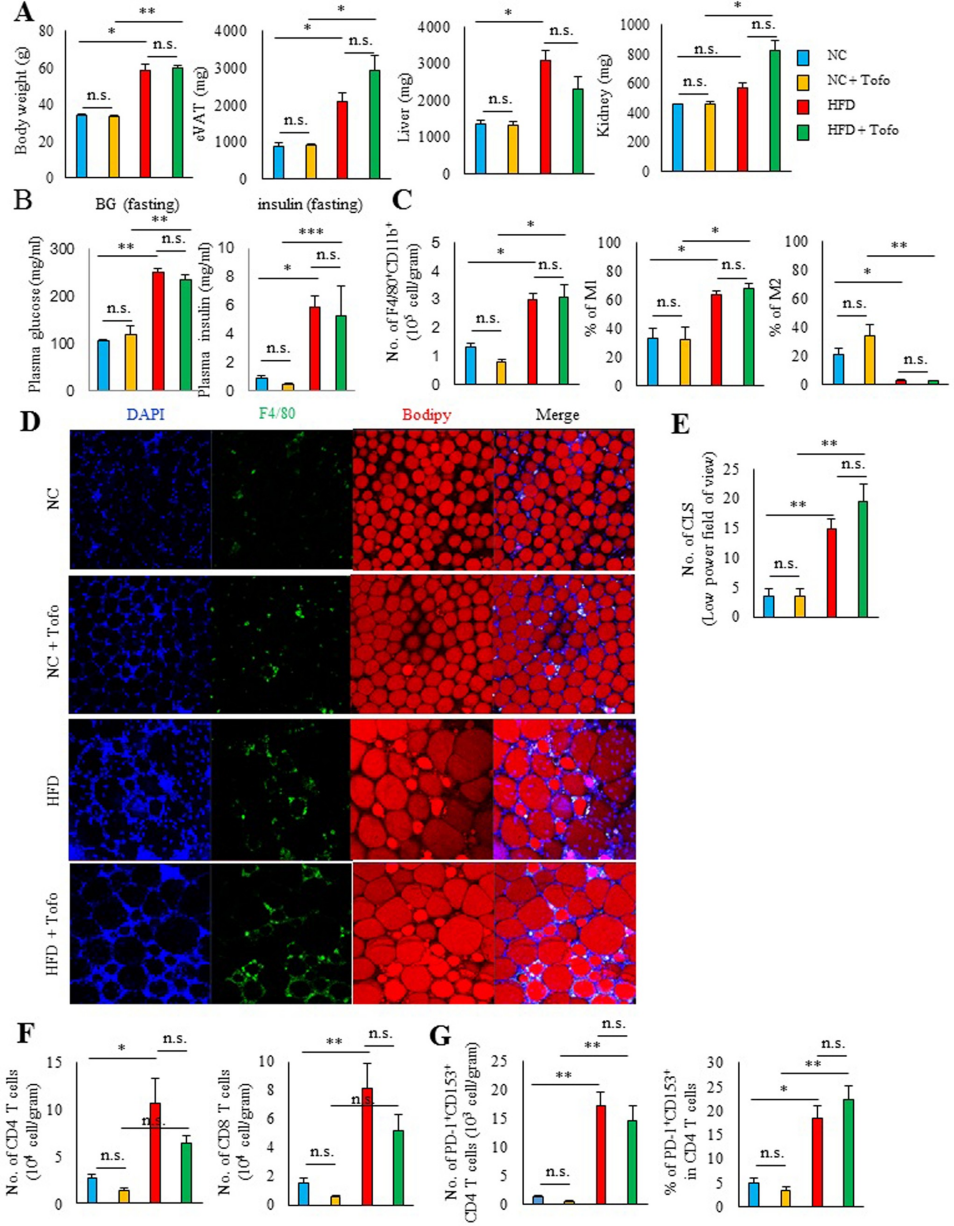
Effect of tofogliflozin on adipose immune cells of obese mice. Mice were fed either HFD or NC with/without tofogliflozin. (A) Body weight, epididymal VAT (eVAT) weight, liver weight, and kidney weight (n = 6 per group). (B) Fasting blood glucose and insulin level. (C) Effect of tofogliflozin on adipose tissue macrophages. Flow cytometric analysis of VAT F4/80^+^CD11b^+^ cells obtained from the indicated mice. Results are expressed as the number of F4/80^+^CD11b^+^ cells per gram of tissue (left). CD11c^+^CD206^-^ cells (middle, M1) and CD11c^-^CD206^+^ cells (right, M2) among F4/80^+^CD11b^+^ cells (n = 6 per group). (D) Histochemical identification of adipocytes (BODIPY, red), F4/80 (green), and nuclei (DAPI, blue) in VAT. (E) Number of crown-like structures in adipose tissue of the indicated mice at low power (n = 6 per group). (F) Flow cytometric analysis of CD4^+^ and CD8^+^ T cells in VAT obtained from the indicated mice. Results are expressed as the number of CD4^+^ and CD8^+^ T cells per gram of tissue (n = 6 per group). (G) Flow cytometric analysis of CD153^+^ PD-1^+^ CD4^+^ T cells in VAT obtained from the indicated mice. Results are expressed as the number of CD153^+^ PD-1^+^ CD4^+^ T cells per gram of tissue (left) or expression of PD-1^+^ CD153^+^ by CD4^+^ T cells (right) (n = 6 per group). *P < 0.05 and **P < 0.01; NS: not significant by the non-parametric Bonferroni test. Data are shown as the mean ± SEM.

**Fig 5:**
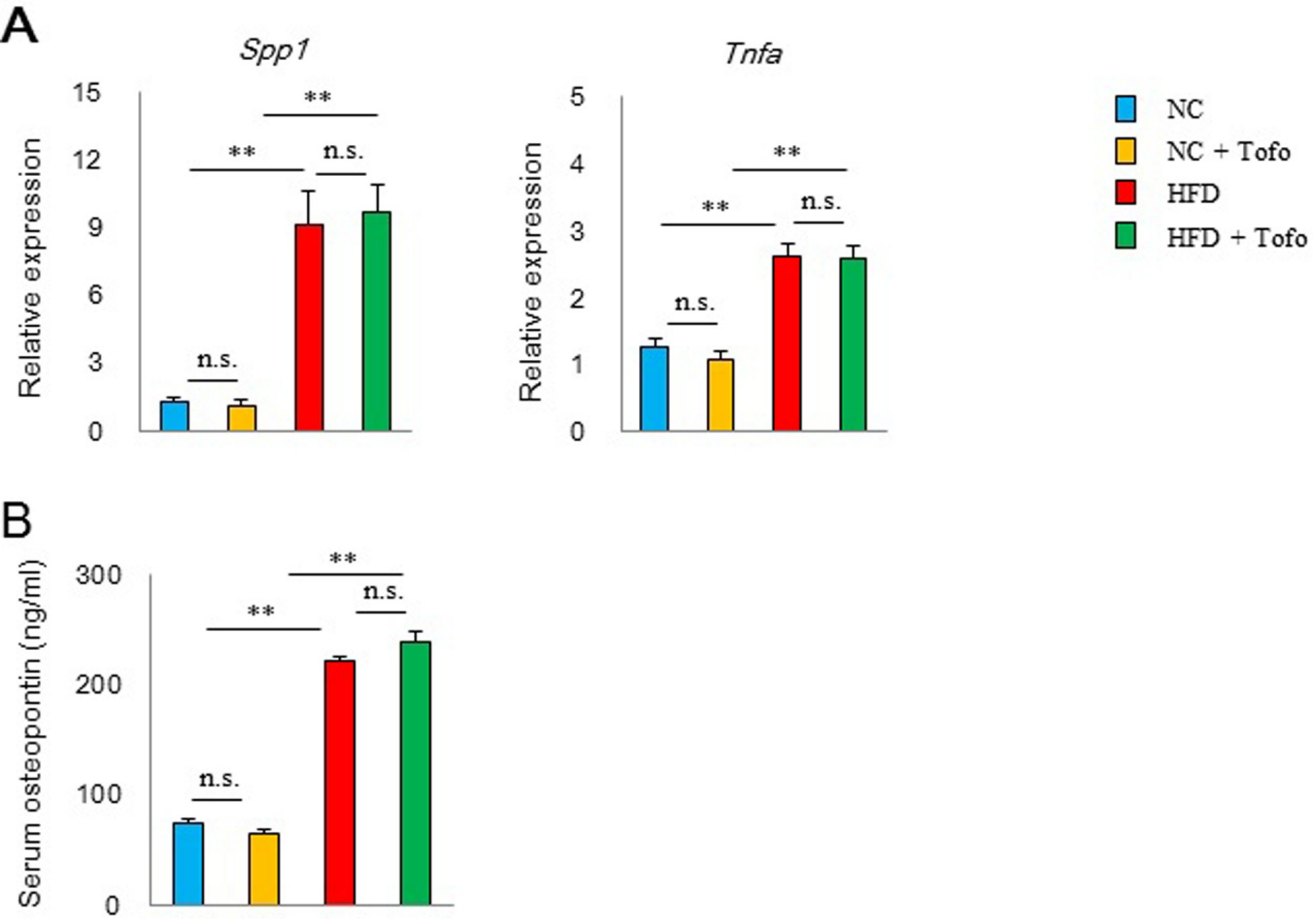
Effect of tofogliflozin on visceral fat inflammation of obese mice. Mice were fed either HFD or NC with/without tofogliflozin. (A) the gene expression of *Spp1* and *Tnfa* in VAT. (B) Serum osteopontin concentration of the indicated mice was assessed by ELISA (n = 7–10) **P < 0.01; NS: not significant by the non-parametric Bonferroni test. Data are shown as the mean ± SEM.

In 38-40^th^ weeks of administration (68–70 weeks of age), body weight, VAT weight, and liver weight were increased in mice receiving the HFD compared with mice switched to NC (Fig 4A). Adding tofogliflozin to the HFD did not affect the body weight, VAT weight, liver weight, kidney weight (Fig 4A) and the fasting blood glucose, insulin level (Fig 4B). In mice on the HFD, prominent macrophage infiltration and crown-like structures were observed in VAT (Fig 4C–4E), with CD11c^+^ M1 macrophages, which induce adipose inflammation [19] [20], being predominant. Massive CD4 ^+^ and CD8 ^+^ lymphocyte infiltration was also observed (Fig 4F), and CD153 ^+^ PD-1^h^CD4 ^+^ T cells (senescence-associated T cells) [8] accounted for 20% of all CD4 T cells (Fig 4G). In parallel with the increase in senescence-associated T cells, the expression of inflammatory genes including *Spp1*and *Tnfa* increased in VAT (Fig 5A), and the blood osteopontin concentration also increased compared to the NC group (Fig 5B). In mice switched to NC, there was only slight macrophage infiltration of VAT and few crown-like structures remained (Fig 4D and 4E). In VAT of the NC group, the portion of CD11c^+^ M 1 macrophage was lower and the proportion of CD206^+^ M 2 macrophage was higher than those of HFD (Fig 4C). Adipose CD4 and CD8 T cells were markedly fewer than those of the HFD group, and senescence-associated T cells showed a significant decrease to only a few percent of all CD4 T cells (Fig 4C–4G). Addition of tofogliflozin to the HFD did not influence on the infiltration of macrophages or T cells and the development of senescent-associated T cells in VAT (Fig 4C–4G). Moreover, there were no significant differences in change the pro-inflammatory gene expression of Spp1 and Tnfa (Fig 5A) in VAT andor blood osteopontin concentration (Fig 5B) between the HFD and the HFD with tofogliflozin groups.

## Discussion

### Effects of weight loss by switching to a control diet

When DIO mice fed the HFD until 30 weeks of age were switched to NC, their weight decreased to the same level as that of mice maintained on NC since weaning [6]. Although VAT shrinks rapidly as the body weight decreases, senescence-associated T cells (CD153^+^ PD-1^+^ CD4^+^ T cells) remain in this fat for at least 2 months after weight loss and continue to produce osteopontin [14]. Osteopontin is so important for T cell survival [21]. Therefore, osteopontin-producing senescence-associated T cells, once they are present, cannot be easily eliminated, and VAT inflammation persists as long as senescence-associated T cells exist. However, the present study showed that senescence-associated T cells disappeared from VAT and chronic inflammation subsided completely with long-term weight reduction (10 months). It is considered that as long as the weight was maintained after weight loss, senescence-associated T cells were not newly produced after changing to NC, and the number of these cells that had ended their lives had decreased. We need more detailed analysis of the mechanism how senescence-associated T cells decrease in VAT in the future. It is reported that sustained weight maintenance after weight loss improved cardiovascular mortality, however rebound exacerbated them in diabetic patients [22]. It is also reported that sustained high concentrations of blood osteopontin are poor prognostic factors such as cardiovascular disease, kidney disease and all-cause mortality [13][23]. The reduction in osteopontin concentration due to weight maintenance after weight loss may contribute to the improvement of prognosis.

Thus, we concluded that weight loss and long-term weight maintenance can eliminate negative legacy of obesity, and which may resulted in the improvement in the beneficial effect.

### Lack of weight loss with tofogliflozin

Among previous investigations into the effect of SGLT2 inhibitors on the body weight in animals receiving an HFD, obvious inhibition of weight gain by SGLT2 inhibitor therapy was only observed when administration was started before body weight reached its peak. This suggests that inhibition of weight gain by SGLT2 inhibitors can be achieved if administration starts almost simultaneously with the HFD at a young age [16][24][25] or when there is a short time on the HFD before administration [26–28]. On the other hand, “weight loss” is less likely if SGLT2 inhibitor therapy is started after body weight has reached its peak on a long-term HFD as in our study. Based on our OGTT data, blood glucose increased gradually after glucose loading in the HFD group and then declined rapidly after reaching a peak. It is therefore considered that glucose-induced insulin secretion by pancreatic beta-cells was maintained and the non-fasting blood glucose was not increased. The insulin level was also high so that lipolysis was unlikely to be prominent. Moreover, the amount of carbohydrate contained in the HFD was so small that the influence of tologliflozin on the UGE was insufficient, and it might lead to no contribution to weight loss. These are considered to be factors related to the lack of weight loss in mice receiving tofogliflozin.

### Lower urinary glucose excretion by tofogliflozin of the HFD group than that of the NC group

Since the HFD contains less carbohydrate compared to NC (26.3 gm% vs. 67.3 gm%), but the total daily food intake is similar (3 g/day), the daily carbohydrate intake of the HFD group was calculated to be half or less of that in the NC group. This may have been related to lower urinary glucose excretion in the HFD with tofogliflozin group compared to NC with tofogliflozin group. There was no marked difference in the non-fasting blood glucose or HbA1c between the HFD group and the NC group, with only fasting blood glucose being higher in the HFD group. Urinary glucose excretion was measured in fed mice and it was not directly affected by the fasting blood glucose level. In the situation where there is no difference in non-fasting blood glucose, it is expected that glucose will be excreted into the urine depending on the amount available when its reabsorption is inhibited.

### Lack of the effect of tofogliflozin on the development of chronic inflammation and senescence-associated T cells in VAT of HFD

Administration of tofogliflozin to mice on the HFD enhanced UGE, but did not reduce the body weight or improve glucose metabolism. These results are consistent with previous report, and this may be due to the compensatory increase in endogenous glucose production in the tofogliflozin group responding to the increased UGE or Ht levels [24]. Among VAT of obese mice fed HFD, various inflammatory cells such as T cells and macrophages are increased, causing chronic inflammation leads to the pathology of diabetes [20]. Since the carbohydrate intake of HFD was so small that obesity due to HFD did not benefit from SGLT2 inhibitor on the inhibition of the development of pro-inflammatory immune cells including senescent-associated T cells and CD11c^+^ M1 macrophages, and which might result in no beneficial effect on the chronic inflammation of VAT and glucose metabolism.

Moreover, we considered the possibility of the influence of compensatory overeating on the effects of tofogliflozin. We observed no significant differences in food intake or the effects on glucose metabolism were noted at some blood sampling point (10–11, and 14^th^ weeks after administration).Food intake tended to increase from 20th week of administration (50 weeks of age) in the HFD with tofogliflozin group compared to the HFD group, however there were no difference of carolie intake per body weight between these groups. Therefore, it is unlikely that an increase in food intake overcame the pharmacological effect of tofogliflozin on glucose metabolism.

Analysis of visceral fat immunity using a model that becomes obese due to overeating such as ob/ob mouse is a future task, however we conclude that tofogliflozin has poor influence on chronic inflammation and the diversity of visceral adipose immunity, at least in a condition in which obesity is caused by diet with low carbohydrate.

### Persistence of albuminuria in the HFD group despite tofogliflozin therapy

It was reported that both albuminuria in association with improvement of hyperglycemia after administration of empagliflozin in Akita mice with type I diabetes [29]. In addition, a renoprotective effect of SGLT2 inhibitor administration was noted in db/db mice, and renal lipid accumulation was also inhibited [30]. Moreover, renoprotective effect (inhibition of mitochondrial injury) was also demonstrated in DIO mice adding ipragliflozin [31].However, it was reported that administration of dapagliflozin for 12 weeks to 12-week old db/db mice increased albuminuria, and glomerular and renal tubular damage showed exacerbation [32]. In this study, the glucose load on the proximal tubules in the HFD group was assumed to be approximately half of that in the NC group. In this situation, SGLT2 inhibitor therapy did not have the effect of resting the proximal tubules [28], inhibiting glomerular hyperfiltration via tubuloglomerular feedback, or improving glucose metabolism due to calorie loss from urinary excretion.

## Conclusion

Chronic inflammation of visceral fat induced by an HFD showed improvement with disappearance of senescence-associated T cells after long-term weight reduction. In our HFD mice with a small carbohydrate load, weight reduction and improvement in glucose metabolism by tofogliflozin were not observed, suggesting that SGLT2 inhibitor therapy is not effective unless there is a certain glucose load. Therefore, it may be difficult to observe the organ-protective effects of SGLT2 inhibitor therapy (inhibition of microalbuminuria, cardiac or renal hypertrophy, chronic inflammation of visceral fat) unless a model is devised in which the SGLT2 inhibitor significantly increases urinary glucose excretion, decreases blood glucose, improves glucose toxicity, and restores endogenous insulin secretion. Whether tofogliflozin could inhibit chronic inflammation of visceral fat in such a model warrants further investigation.
